# C1q and central nervous system disorders

**DOI:** 10.3389/fimmu.2023.1145649

**Published:** 2023-03-23

**Authors:** Wenjie Zhang, Yuan Chen, Hui Pei

**Affiliations:** ^1^ Department of Emergency Intensive Care Unit, the First Affiliated Hospital of Zhengzhou University, Zhengzhou, China; ^2^ Department of General Practice, Xingyang Sishui Central Health Center, Zhengzhou, China; ^3^ Department of Neurology, the First Affiliated Hospital of Zhengzhou University, Zhengzhou, China

**Keywords:** c1q, central nervous system, neurodevelopment, degenerative diseases, inflammatory diseases, glioma

## Abstract

C1q is a crucial component of the complement system, which is activated through the classical pathway to perform non-specific immune functions, serving as the first line of defense against pathogens. C1q can also bind to specific receptors to carry out immune and other functions, playing a vital role in maintaining immune homeostasis and normal physiological functions. In the developing central nervous system (CNS), C1q functions in synapse formation and pruning, serving as a key player in the development and homeostasis of neuronal networks in the CNS. C1q has a close relationship with microglia and astrocytes, and under their influence, C1q may contribute to the development of CNS disorders. Furthermore, C1q can also have independent effects on neurological disorders, producing either beneficial or detrimental outcomes. Most of the evidence for these functions comes from animal models, with some also from human specimen studies. C1q is now emerging as a promising target for the treatment of a variety of diseases, and clinical trials are already underway for CNS disorders. This article highlights the role of C1q in CNS diseases, offering new directions for the diagnosis and treatment of these conditions.

## 1 Introduction

**Figure 1:**
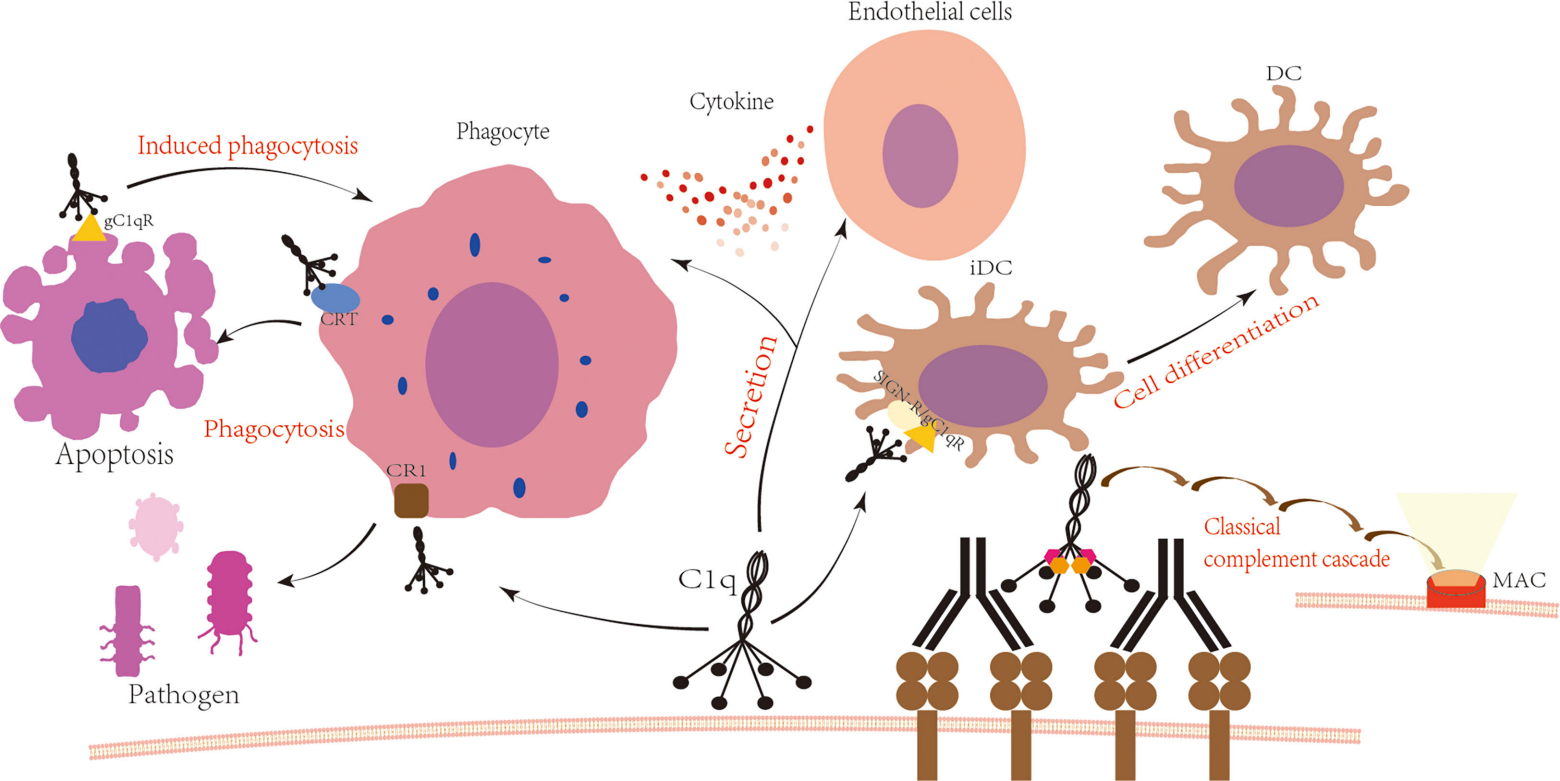
C1q is involved in the immune process. Upon antigen-antibody binding, C1q is activated and subsequently activates C1r and C1s, leading to the formation of C3 convertase C4b2a, C5 convertase C4b2a3b, and ultimately the formation of the membrane attack complex with the involvement of C6 to C9. The globular domain or collagen-like tail of C1q binds to cell surface receptors to produce various physiological effects. The globular domain of C1q binds to SIGN/gC1qR, which regulates the differentiation of immature dendritic cells (3, 4). The tail of C1q binds to the phagocyte surface receptor CR1, promoting the phagocytosis of pathogens (5). Additionally, the globular domain of C1q interacts with the phagocyte surface receptor CRT to promote phagocytosis of apoptotic cells, while binding to gC1qR on the surface of apoptotic cells induces phagocytosis (6, 7). Furthermore, C1q interacts with phagocytes and endothelial cells, increasing cytokine secretion (8, 9).

The complement system is a sophisticated protein response system consisting of over 30 components that are present in serum, tissue fluids, and cell surfaces. It possesses a complex regulatory mechanism (1). These components exist in a non-activated form in body fluids and are activated through a cascade of enzymatic reactions that produce various biological effects. There are three complement activation pathways, each with a common end-reaction process (2). The classical pathway involves a cascade of enzymatic reactions in which activators, mainly IgG and IgM bound to antigen, bind to C1q and sequentially activate C1r, C1s, C2, and C3, forming C3 convertase (C4b2a) and C5 convertase (C4b2a3b), ultimately leading to the formation of the C5b6789n complex, the membrane attack complex (MAC), at the cell membrane (**Figure 1**). The alternative pathway directly activates C3 by microorganisms or exogenous xenobiotics to form C3 convertase (C3bBbP) and C5 convertase (C3bBb3b), with the involvement of B-factor, D-factor, and P-factor. The lectin pathway, also known as the MBL pathway, involves a cascade of enzymatic reactions in which mannose-binding lectin (MBL) and ficolin (FCN) in plasma directly recognize the glycan structure on the surface of the pathogen, sequentially activating MBL-associated serine protease (MASP), C4, C2, and C3, ultimately forming the same C3 convertase and C5 convertase as in the classical pathway.

C1q is a crucial component of the complement system, produced mainly by myeloid cells such as monocytes, macrophages, and immature dendritic cells. It is composed of three genes (C1q A, B, and C) that encode proteins with a collagen-like region and a globular domain. The A, B, and C chains are assembled into trimers, and six trimers form a hexamer with a total of 18 polypeptide chains. The resulting complex molecule has multiple interaction sites on C1q. The crystallographic model revealed that the binding site of the immunoglobulin to C1q is located on the outer surface of the head, while the apoptotic cells bind on the inner surface of the sphere (10). C1q can act as a biomolecule recognized by pathogen-associated molecular patterns (PAMPs) and danger-associated molecular patterns (DAMPs) to perform immune functions (11). It has an essential role in removing foreign substances and pathogens, as well as maintaining internal stability. C1q is well known for its involvement in the classical activation pathway of complement. When the globular head of C1q binds to the Fc segment of IgM or IgG in the immune complex, the C1q conformation changes, leading to the sequential activation of C1r and C1s, initiating the classical activation pathway of complement. C1q has four main types of receptors, including Clq receptor for phagocytosis enhancement (ClqRp), complement receptor 1 (CR1), calreticulin (CRT), and C1q globular head binding protein (gClqbp) (12). By interacting with supracellular receptors, C1q stimulates variety of cells such as phagocytes and endothelial cells to secrete a variety of cytokines, exacerbating local inflammatory responses (8, 9, 11). Recent research has shown that the interaction of C-type lectin SIGN-R and C1q head can promote the maturation and differentiation of immature DCs(iDCs) cells (3). Additionally, the regulation of DC differentiation and function is through C1q binding to cell surface SIGN and gC1qR-specific molecules on the DC plasma membrane to form a trimolecular complex (4). In pneumococci, the SIGN-R1 receptor on macrophages binds directly to C1q replacing the Ig normally used in the classical pathway, forming C3 convertase, subsequently activating the rest of the pathway (13). CR1 binds to C1q and is involved in phagocytic activity (5), CR1 is widely expressed and is also found on CNS microglia (14). CRT binds to the C1q globular domain, enhances phagocytosis of apoptotic cells, and triggers an immunogenic response (6, 15). C1qRp binds to the C1q collagen tail and globular head sites and induces phagocytosis of apoptotic cells (7). C1q also has anti-cancer effects through immunosurveillance (16, 17), Antigen-presenting cancer-associated fibroblasts (apCAFs) in lung malignancies produce C1q, which binds to T cells C1qbp to inhibit T cell apoptosis and promote tumor killing by immune cells (18). The presence of C1r2s2 increases the stability of C1q-IgG and has a positive effect on the elimination of foreign substances and tumor cells (19). Apart from normal physiological immune responses such as the removal of foreign substances and abnormal cells, C1q also has an important impact on the development of other diseases. C1q can act in conjunction with ApoE to cause atherosclerotic plaque formation (20), can be present alone to participate in the early development of atherosclerosis (21). C1q complexes with vWF increase platelet rolling and adhesion and promote hemostasis (22). C1q binds to discoidal domain receptor 2 (DDR2) to regulate metalloproteinase expression for wound healing due to its specific collagen-like structure (23). The structure of the C1q collagen region binds to the sequence of fibronectin-binding protein B (PfbB) on the surface of Streptococcus pneumoniae and promotes the adhesion of Streptococcus pneumoniae to host cells and participates in the process of pneumococcal infection (24). Complement activation is well documented in the progression of autoimmune diseases and is especially important in the development, diagnosis and prognostic monitoring of lupus nephritis (25, 26). C1q blocks aberrant effector CD8+ T cell responses by recognizing activated CD8+ T cells through globular structural domains and affecting their mitochondrial metabolism, and such aberrant effector CD8+ T cells are known to be involved in the development of systemic lupus erythematosus (SLE) (27). A recent study by Zheng et al. in Behçet’s disease showed a significant pro-inflammatory effect of monocytes with high expression of C1q (C1qhi) in the cell membrane by single cell RNA sequencing (scRNA-seq) technology (28). This highlights the importance of understanding the role of C1q in autoimmune diseases and other inflammatory.

## 2 Neural development

A growing body of research has identified C1q as a key substance involved in neuronal network development and homeostasis within the central nervous system. Specifically, C1q serves as a pruning function during synapse development and synaptogenesis (29, 30). Microglia are extremely delicate and strict on synaptic pruning processes in the nervous system, and C1q and C3 may act as phagocytic signals for microglia-dependent phagocytosis of synapses (31, 32). Multiple EGF-like domains 10 (Megf10), a class F scavenger receptor expressed on astrocytes, binds to C1q with high affinity and induces phagocytosis of apoptotic cells (33). In developing mice, exposure to phosphatidylserine (PS) functions as a neuronal “eat-me” signal involved in C1q-mediated pruning with the microglia receptor TREM2 (34). PS is not exposed on the intact cell surface, but is exposed upon apoptosis (35). In a study of visual neurons in mice, C1qa was found to be critical in the formation of synapses between retinal ganglion neurons and dorsal lateral geniculate nucleus neurons in the visual thalamus, but C1qa did not affect synaptic density and microglia phagocytosis in the developing visual cortex (36). It indicates that different complement components are inconsistent at different developmental periods and regions, and their complex relationships need further study.

**Figure 2:**
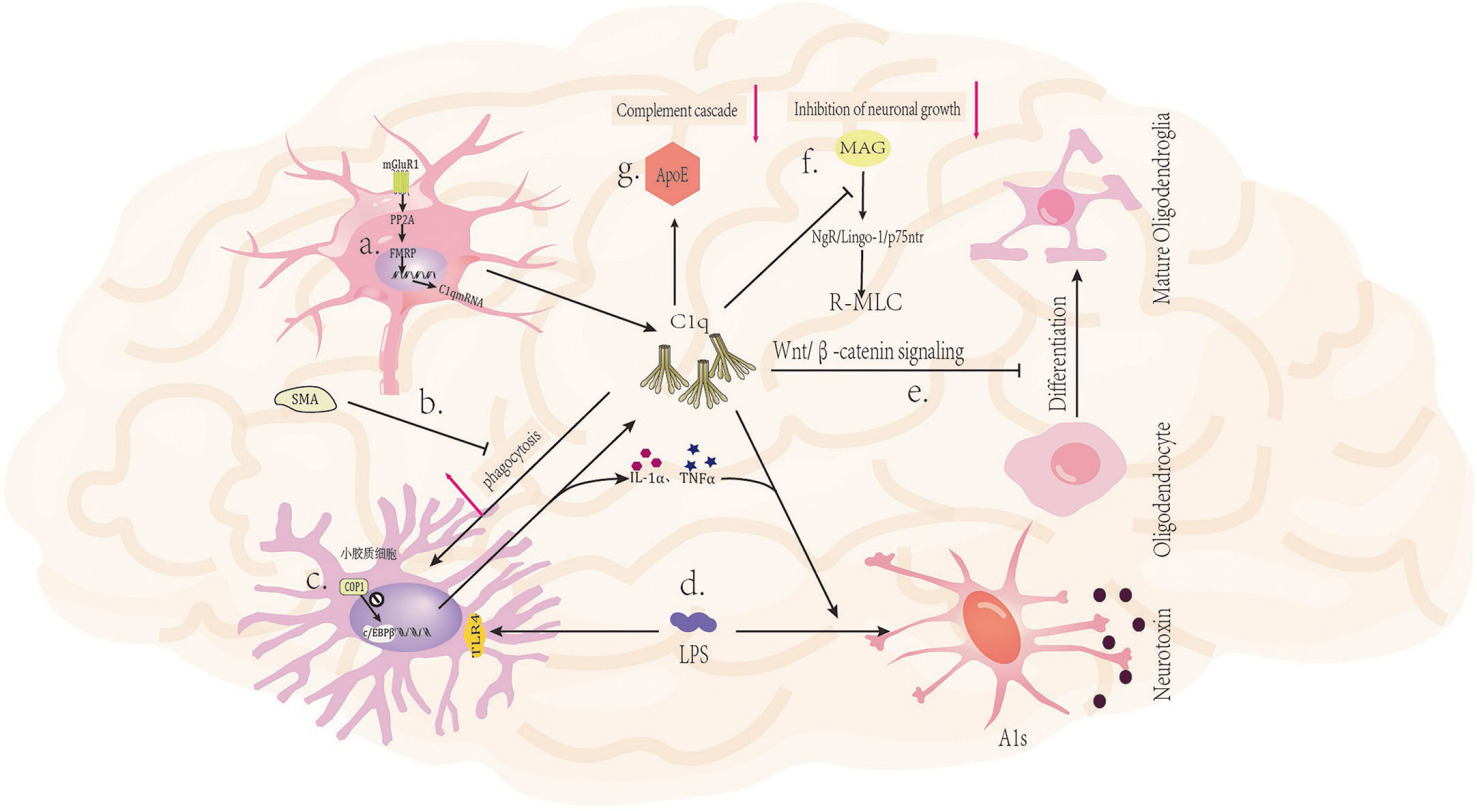
Mechanism of C1q in nervous system diseases. **(A)** Activation of glutamatergic neuronal mGluR1 receptors leads to increased expression of C1q mRNA (43). **(B)** The mGluR5 silent alteration modulator (SAM) inhibits C1q-mediated microglial phagocytosis (44). **(C)** The ubiquitin ligase COP1 regulates the transcription process of microglial inflammatory genes by controlling CCAAT/enhancer binding protein β (c/EBPβ) (45). **(D)** LPS (lipopolysaccharide) can bind to TLR4 receptors on microglia, triggering the secretion of C1q, Il-1α, and TNFα by microglia, and inducing A1 in astrocytes (46). **(E)** C1q prevents the differentiation of oligodendrocyte progenitor cells (OPCs) into mature oligodendrocytes by inhibiting the Wnt/β-catenin signaling pathway (47). **(F)** C1q interacts with MAG to reduce MAG inhibitory effect on neuronal signals, thereby promoting axonal growth (48). **(G)** The binding of ApoE to C1q inhibits the initiation of the classical complement cascade reaction (20).

The role of the complement pathway in the brain is complex, and there is now evidence that complement plays an important role in neurological disorders in adulthood (37, 38). Complement activation may contribute to neurological disorders through the binding of C3b (iC3b)-CR3 to phagocytes for neuronal damage (39), or through the destruction of neurons by binding to specific allergenic toxin receptors on local glial cells (40). C1q on the one hand gives rise to the classical complement pathway and on the other hand is involved in several functions that may be independent of the complement cascade, including regulation of synaptic pruning (31, 32), protection against neurotoxicity (41) and promotion of angiogenesis (42). In neurological disorders, it may have many deleterious effects as well as some beneficial effects, while evidence for these effects primarily comes from animal models, there is also some evidence from human specimen studies. Overall, an in-depth understanding of the effects of C1q on the nervous system’s development and function can provide insight into the role of C1q in neurological disorders and may offer potential diagnostic and treatment strategies (**Figure 2**). This article revolves around a review of recent research advances on C1q in recent years, which are expected to provide new ideas for the diagnosis and treatment of neurological disorders.

## 3 Relationship between C1q and microglia and astrocytes

C1q, which is produced by microglia, astrocytes, and neurons, plays different roles in the CNS. Microglia and astrocytes are crucial cells in CNS disorders related to C1q. Astrocytes, as the most abundant glial cells, are a vital component of the blood-brain barrier and exhibit incredible complexity in different brain regions (49, 50). Microglia, as the brain’s resident macrophages, participate in various neuroimmune and non-immune processes (51). Microglia and astrocytes have important roles in neural repair, growth, homeostasis, and interneuronal signaling. Abnormalities in both will result in the development of a variety of diseases. During nervous system development, microglia interact with C1q and C3 to regulate synapse pruning. When stimulated by foreign C1q, microglia produce C1q to activate more microglia, creating a positive feedback loop and promoting inflammation (52). However, complement activation can also mediate pathological synaptic loss in microglia during aging, leading to degenerative diseases (31). Astrocytes can respond to various stimuli and undergo changes in gene expression, function, and morphology, resulting in “reactive astrocytes” that can have both protective and harmful effects on neurons (53). Inflammatory mediators secreted by microglia, such as IL-1α, C1q, and TNF, can induce astrocytes to become reactive astrocytes (A1s) (54). The pro-inflammatory factors TNF, IL1α and complement pathway component C1q are able to induce a deleterious response state in astrocytes that are distinct from healthy astrocytes (46). A1s are cells that have lost their typical astrocyte functions (promoting neuronal survival and growth, facilitating synapse formation, phagocytosis of synapses and myelin debris, etc.) (55–57). Studies with rodent and human astrocytes suggest that activated microglia are not sufficient to kill neurons, but rather induce A1s by inducing the secretion of IL-1α, TNFα, and C1q together, and that A1s are capable of secreting neurotoxins and releasing a variety of complement components that denature synapses, leading to neural deformation and death (46, 58). A1s can denature synapses, leading to neural deformation and death, and are implicated in several neurodegenerative diseases such as Alzheimer’s, Huntington’s, Parkinson’s, Amyotrophic Lateral Sclerosis, and Multiple sclerosis. Transcriptomic analysis reveals that the transcriptional profiles of reactive astrocytes (A1s) and inflammatory microglia (MIMS) overlap with those of other neurodegenerative glial cells. C1q has been identified as a crucial mediator of A1s and MIMS activation (59), which will be further analyzed in subsequent sections.

## 4 Relationship to degenerative diseases in the central nervous system

Neurodegenerative diseases are a group of chronic and progressive diseases that cause damage to tissues such as the central nervous system or peripheral nervous system, and their underlying mechanisms are not yet fully understood. These diseases include Alzheimer’s disease (AD), Amyotrophic Lateral Sclerosis (ALS), Parkinson’s disease (PD), among others. Recent studies utilizing single-cell RNA sequencing have revealed significant differences in gene expression in patient cells, and have identified the accumulation of complement C1q as a central factor underlying most proteotype-related functional synaptic dysfunction (60). C1q plays a crucial role in the development of degenerative diseases, and the following section provides a brief overview of the relationship between C1q and the aforementioned diseases in recent years (**Table 1**).

**Table 1:** The effect of C1q in neurological disorders.

Disease	Object	Research conclusion	C1q effect	References
Alzheimer’s disease	CSF of AD patients	Elevated inflammatory markers such as C1q in pre dementia	Detrimental	(61)
	*C1qa–/–*, *C6–/–*,*Cd59a–/–*, *AppNL–G–F* and 3xTg-AD mice	C1q increased significantly in the nervous system of AD mice	Detrimental	(62)
	Choroid plexus in AD patients	C1q ApoE complex may play a physiological role in normal brain homeostasis	Beneficial	(20)
	Sprague Dawley rat, *TNFα* -/-,*C1q(a*) –/–,*Il-1α –/– TNFα* –/– and *Il-1α –/– TNFα* –/– *C1q* –/– mice	C1q induced A1s	Detrimental	(46)
	Cop1fl/fl,Cebpb-/-,Rosa26-CreERT2,Cx3cr1-CreERT2-eYFP, and Tau(P301S) mouse	C1q activation caused by COP1 inactivation	Detrimental	(45)
	Sprague-Dawley rats,Tg-APPsw/PSEN1DE9 (APP/PS1) mice and Metabotropic glutamate receptor 1 mutant mice	MGluR1 increases the expression of C1q mRNA and enhances the phagocytosis of microglia	Detrimental	(43)
	MAPT gene KI AD mouse model and a TG amyloidogenic mouse model	SAM Prevents Synaptic Localization of C1q and Synaptic Phagocytosis of Microglia in AD Mice	Detrimental	(44)
Amyotrophic Lateral Sclerosis	Spinal cord and motor cortex in ALS	C1q deposition	Detrimental	(63)
	SOD1G93A mice and ALS necropsy	C1q Deposition on the Moving End Plate	Detrimental	(64)
	hiPSC- astrocytes and SOD1 G93A in mice	C1q as inducer	Detrimental	(65)
	SOD1G37R ALS mice and *C1qa* KO mice	C1q does not contribute significantly in the pathogenesis of ALS	Neutral	(66)
Parkinson	PD patients, healthy controls serum	4R-Tauopathies complement levels are lower than those of PD patients and HC patients, which is useful for disease differentiation	——	(67)
	MPTP mouse model	C1q may act here as a mediator of extracellular debris removal by microglia	Beneficial	(68)
	The nigrostriatal in PD and control cases	C1q mediates microglia clearance of extracellular debris	Beneficial	(69)
	MSA donor brain samples	α-syn can be recognized by C1q, which initiates the classical complement pathway and forms a membrane attack complex to induce cell death	Detrimental	(70)
Multiple Sclerosis	MS Autopsy	Increased expression of C1qA	Detrimental	(71)
	MS patient’s brain	C1q labeled synapse	Detrimental	(72)
	MS Dead Seahorse Area	C1q impairs cognitive function	Detrimental	(73)
	CPZ mice	C1q activation of MIMS	Detrimental	(59)
	Plasma from MS patients	C1q participation in MS prominently lost	Detrimental	(74)
	crEAE mouse model	C1q continues to increase	Detrimental	(75)
	EAE mice, C1q cKO mice	Targeted C1q therapy to mitigate disease progression	Detrimental	(76)
	CPZ mice	C1q prevents oligodendrocyte progenitor cells from differentiating into mature oligodendrocytes	Detrimental	(47)
Huntington’s disease	HD patients and control group	Complement C mRNA expression in the brain of HD patients	Detrimental	(77)
	NPA induced in Wistar rats	Cytokine IL-1α, TNFα and C1q expression in the striatum, hippocampus and cerebellum are upregulated, further causing neuronal damage	Detrimental	(78)
	striatum in HD patients and controls	Astrocyte proliferation and microglia with marked hyperplasia and increased complement expression	Detrimental	(79)
Nerve injury	C57BL/6J mice, BCKO mice, *C3 –/–*mice	C1q deposition at the site of axonal pathology and demyelination	Detrimental	(80)
	BUB/BnJ mice, C1q KO mice	C1q can mediate oligodendrocyte death	Detrimental	(81)
	C57BL/6J mice, TBI mouse model	C1q causes a strong follow-up reaction to TBI	Detrimental	(82)
	mTBI mouse model	C1q causes further damage to the thalamus	Detrimental	(83)
	C57B6/J mice, cortical impact or sham surgeries	C1q impairs cognitive function	Detrimental	(84)
	Cerebrospinal fluid in TBI patients	C1q subunit B is present in released microvesicles and exosomes (MV/E)	Detrimental	(85)
	Serum of TBI patients	C1q was significantly correlated with GCS score and Rotterdam CT classification	Detrimental	(86)
	Sprague Dawley rat、C1q KO BUB/BnJ mice	C1q decreases the effect of MAG on neuronal inhibitory signals and thus promotes axonal growth	Beneficial	(48)
	C57BL/6Jmice, 129Smice,*C1q A chain* KOmice,*C3* KOmice,*CD11b* KOmice	C1q has a reparative role in nerve injury	Beneficial	(87)
Guillain-Barré syndrome	EAN-induced Lewis rat model	Antibody binding to C1q causes complement activation and exacerbates demyelination	Detrimental	(88)
Gliomas	Primary isolation and cell culture of human brain	RXFP1 - CTRP8 may also promote the increase of glioblastoma migration through STAT3 signal mediated actin cytoskeleton remodeling and fibropodia formation	Detrimental	(89)
	GBM tumor tissue	Deposition of C1q and C3 was observed in the tumor tissue	Detrimental	(90)
	GEPIA Database	CTRP1 can regulate the expression of CCL2 to promote tumor progression	Detrimental	(91)
	Onomine database, UALCAN and CGGA database analysis	There is a positive correlation between the level of C1q expression and different grades of glioma	Detrimental	(92)

### 4.1 Alzheimer’s disease (AD)

Alzheimer’s disease (AD) is a common neurodegenerative disorder that affects the elderly, but its underlying mechanisms are still not fully understood. The cognitive impairment observed in AD is associated with synaptic loss and morphological changes in the brain (93). A study of cerebrospinal fluid from patients with AD pathology (beta-amyloid (Ab) and tau subtypes) but not yet symptomatic or in pre-dementia found elevated YKL-40, sTREM2, sAXL, sTyro3, MIF, complement factors C1q, C4 and H, ferritin, and ApoE inflammatory markers (61). C1q has been found to induce excessive phagocytosis and synapse clearance by microglia, leading to synapse loss in AD patients (93, 94). Carpanini et al. conducted experiments on AD model mice and discovered complement dysregulation, with C1q significantly increased in the nervous system of AD mice (62). Yin et al. studied the inflammatory response in the choroid plexus (ChP) of AD patients and found a specific interaction between ApoE and C1q. They observed that the high-affinity binding of ApoE to C1q inhibits the initiation phase of the classical complement cascade reaction (CCC), with the ApoE isoform binding to the activated form of C1q in a Ca2+-dependent manner (20). These findings suggest a close relationship between C1q and neurodegenerative lesions in AD. Liddelow et al. used a mouse model of AD and found that reactive astrocyte A1 (A1s) plays an important role in AD progression (46). A1s-reactive astrocytes can also cause neurological damage *via* astrocyte-derived exosomes (ADE) and deliver them to other CNS cells *via* ADE (95). Another study found that the transcription factor CCAAT/enhancer binding protein β (c/EBPβ) promotes inflammatory gene transcription in microglia, and that microglia c/EBPβ expression can be regulated by the ubiquitin ligase COP1. Microglia knocked out of COP1 produce c/EBPβ-dependent neurotoxicity mainly through C1q activation of the classical complement pathway. This suggests that the activation of C1q caused by COP1 inactivation plays an important role in AD progression (45). Previous reports have found a strong link between glutamate and the development of AD, so how is C1q produced and regulated in the brain, and what is the link between it and glutamate? Mouse astrocyte glutamate transporter 1 (GLT1) maintains intersynaptic glutamate concentration homeostasis by removing glutamate from the synaptic gap *via* active transport *via* H^+^-K^+^ or Na^+^-K^+^ pumps or by synthesizing glutamine for reuse *via* glutamine synthetase. This suggests that abnormal function or reduced number of the astrocyte glutamate transporter GLT1 may be responsible for the occurrence of AD (96). Bie et al. (43) concluded from their mouse model studies that metabotropic glutamate receptor 1 (mGluR1), when induced by amyloid, activates mGluR protein phosphatase 2A (PP2A), which causes dephosphorylation of fragile X messenger ribonucleoprotein (FMRP), resulting in increased expression of C1qmRNA and enhanced phagocytosis of microglia. Spurrier et al. confirmed this mechanism in the treatment of aged AD mice with metabotropic glutamate receptor 5 (mGluR5) silent alteration modulator (SAM). SAM restored synaptic density and prevented synaptic localization of C1q and synaptic phagocytosis of AD mice by microglia. It also prevented abnormal synaptic signaling induced by β-amyloid oligomers, while maintaining physiological glutamate responses and reducing the accumulation of phospho-TAU. This suggests the possibility of treating AD specifically by targeting mGluR5 for modulation (44).

### 4.2 Amyotrophic lateral sclerosis (ALS)

ALS is the most common adult motor neuron disease, characterized by the progressive loss of upper and lower motor neurons leading to muscle atrophy and eventual death (97). The mechanism underlying the development of ALS is still unclear, and mutations in approximately 30 genes have been identified as causative factors of ALS, including C9orf72, SOD1, FUS, TARDBP, and VCP (98). While most of ALS cases are sporadic, familial cases are also present, with mutations in the gene encoding copper-zinc superoxide dismutase-1 (SOD-1) being more common. As such, many studies on ALS have been conducted on mice with SOD1 mutations. Ferraiuolo et al. used laser capture microdissection and microarray to analyze motor neuron changes in SOD1 G93A mice, revealing significant transcriptional repression, metabolic function decline, upregulation of complement components, and increased expression of cell cycle proteins involved in the cell cycle during the late stage of the disease, indicating the crucial role of complement components in ALS development (99). In ALS, complement components have been found at multiple nerve sites, with deposition of C1q and C4 observed in both the spinal cord and motor cortex of ALS patients, along with higher levels of inflammatory response and microglia activation in patients with rapidly developing ALS (63, 99). Bahia El Idrissi et al. studied immunofluorescence staining of SOD1G93A gastrocnemius and ALS donor intercostal muscle tissue and found that deposition of complement activation products C3/C3b and C1q in the motor endplates during the early stages of ALS symptoms. C1q deposition on the motor endplates of ALS donor intercostal muscles in the SOD1G93A mouse model was detected before the appearance of clinical signs, indicating that complement activation is an early event. C1q immunoreactivity is present in most intercostal muscle tissues of ALS donors, in addition to C1q deposition on motor nerve terminals and terminal Schwann cells of ALS donor intercostal muscles. The study also explored regulatory factors such as CD55 and CD59, which protect tissues from complement system attack and could provide new therapeutic approaches for ALS (64). C1q not only destroys neurons *via* the classical complement system, but also acts as an inducer of reactive astrocytes, as has been briefly suggested above in AD patients. Among the damaged astrocyte mechanisms, damaged astrocyte glutamate uptake was found to cause excitotoxicity and has been suggested to play an important role in motor neuron hyperexcitability and death in ALS (100). In the ALS mouse model astrocytes were compared to A1s astrocytes with respect to glutamate uptake and subsequent toxic response and there was a great similarity. As seen by gene expression analysis of ALS astrocytes versus protective astrocytes, multiple genes appear opposite (65), signaling pathway analysis revealed that inflammatory pathways such as JAK-STAT, NF-kB and TNF are significantly increased in mouse ALS models, but C1q is able to inhibit the activation of these inflammatory pathways in phagocytes (101–103). Peng showed that TDP-43 is required to maintain the protective properties of astrocytes, and TDP-43-deficient astrocytes exhibited increased immunoreactivity but did not affect the proliferation of astrocytes or microglia. At the transcriptome level, TDP-43-deficient astrocytes resembled A1-responsive astrocytes and induced increased C1q expression in microglia (104). Interestingly, Lobsiger et al. found that the induction of complement pathway activation by C1q did not significantly contribute to the ALS pathogenesis in SOD1 G37R mutant mice (66). The role of C1q in ALS has been recognized by most researchers, and although some studies are inconsistent, we still cannot deny the important role of C1q in ALS, a disease that requires continued efforts to explore and study to find more treatments. Clinical trials about C1q inhibitors in ALS have emerged(NCT04569435)(EUCTR2021-000325-26-FR) (**Table 2**).

**Table 2:** Anti-C1q clinical trials on neurological disorders.

Identifier	Recruiting status	Study subjects	Age	Country	Sample size	Intervention	Primary outcome	Access link
NCT04514367	Completed	Hunting’s Disease	≥18 years	United States	28	ANX005	Safety and tolerability of intravenous ANX005 administered for up to 22 weeks in subjects with, or at risk for, manifest Huntington’s Disease	https://clinicaltrials.gov/ct2/show/NCT04514367
NCT04035135	Completed	Guillain-Barré Syndrome	≥18 years	Bangladesh, Denmark	14	ANX005 and IVIg	Pharmacokinetics of ANX005 when administered in combination with IVIg	https://clinicaltrials.gov/ct2/show/study/NCT04035135
NCT04569435	Recruiting	Amyotrophic Lateral Sclerosis	≥18 years	Canada, United States	24	ANX005 IV Infusion	Number of Participants Who Experienced Treatment-Emergent Adverse Events	https://clinicaltrials.gov/ct2/show/study/NCT04569435
NCT04701164	Recruiting	Guillain-Barre Syndrome	≥18 years	National Institute of Neurosciences and Hospital (NINS) Bangladesh	180	ANX005 and Placebo	1.GBS Disability Score (GBS-DS) 2.Number of Participants with Adverse Events	https://clinicaltrials.gov/ct2/show/NCT04701164
EUCTR2021-000325-26-FR	Authorised	Amyotrophic Lateral Scelrosis (ALS)	≥18 years	EUCTR	25	ANX005	Main Objective: To assess the safety and tolerability of ANX005 administered for up to 12 weeks in subjects with ALS Secondary Objective : To assess the pharmacodynamic (PD) effects of ANX005 in blood and CSF through the assessment of NfL and pNFH	https://trialsearch.who.int/Trial2.aspx?TrialID=EUCTR2021-000325-26-FR
NCT04489160	Recruiting	Traumatic Brain Injury	≥18 years and < 65 years	Netherlands	106	C1 Inhibitor and Placebo	1. Therapy Intensity Level (TIL) Scale 2. Glasgow Outcome Scale Extended (GOSE) 3. Complication rate	https://www.clinicaltrials.gov/ct2/show/NCT04489160?term=C1&cond=Traumatic+Brain+Injury&draw=2&rank=1

### 4.3 Parkinson’s disease (PD)

PD is the most prevalent movement disorder of the nervous system and the second most common neurodegenerative disease after AD. The lesions are found in the substantia nigra-striatal area and are characterized by the loss of nigrostriatal neurons, striatal dopaminergic deficiency, and the buildup of alpha-synuclein (α-syn) in intraneural inclusions (105). The incidence of PD tends to increase with age, which is significantly associated with age (106). Initially, PD was thought to be a movement disorder without dementia, with major symptoms in the motor system such as bradykinesia (slow movement), rigidity, and resting tremor. However, with the growing awareness of Parkinson’s disease, PD also affects other extrapyramidal dopaminergic, cholinergic, and serotonergic bundles, leading to non-motor symptoms, including loss of smell, sleep disturbances, and constipation, as well as cognitive and psychiatric symptoms, such as dementia and depression (107). Cognitive impairment is six times more prevalent in individuals with PD than in the healthy population (108). It is one of the most significant non-motor manifestations of PD, and cognitive impairment can significantly impair the quality of life and function of PD patients, with the majority of patients developing dementia within 20 years of diagnosis (109). The pathogenesis of PD is still unknown, and clinical data suggest that PD has a genetic origin, with mutated genes including those encoding alpha-synuclein, DJ-1, PINK, LRRK2, and others (110). Numerous studies have highlighted the involvement of both innate and adaptive immune systems in the development of PD (111, 112). Clinical studies have demonstrated that non-steroidal anti-inflammatory drugs (NSAIDs), including ibuprofen (non-aspirin), may reduce the risk of PD and have a protective effect, particularly in long-term, regular users (113, 114). The difficulty in distinguishing early PD from some Four-repeat (4R-) Tauopathies arises from the lack of specificity in clinical presentation. To address this issue, Khosousi investigated serum C1q and C3 levels and found that 4R-Tauopathies had lower levels of complement compared to PD patients and healthy controls (67). Mice models of nigrostriatal pathway injury induced by MPTP have confirmed microglia activation and increased expression of C1q in the nigrostriatal system. However, in the subchronic MPTP model, C1q may act as a mediator of extracellular debris removal by microglia and did not affect nigrostriatal injury (68). These findings were also confirmed in the nigrostriatal SNc in the Depboylu study of PD and control cases (69). PD is characterized by the accumulation of alpha-synuclein (α-syn) in intracellular Lewy bodies (115), α-syn can activate the classical complement pathway by acting at an early step of the complement cascade. α-syn is secreted extracellularly through the formation of transmembrane pores or released as vesicles to bind to cell surface receptors (115, 116). α-syn can be recognized by C1q, which initiates the classical complement pathway and forms a membrane attack complex to induce cell death (70). The relationship between α-syn and the innate immune system could open new avenues for PD treatment.

### 4.4 Multiple sclerosis (MS)

MS is a chronic autoimmune demyelinating disease of the central nervous system, characterized by lesions in the white matter, gray matter, brainstem, spinal cord, and optic nerve (117). Pathologically, the disease is associated with increased demyelination of white matter, inflammatory response, and gliosis (117, 118). Demyelination is not unique to the white matter, but also involves the gray matter (119, 120). The disease is characterized by recurrent episodes of inflammatory demyelination, which can lead to neurodegeneration, accompanied by a relapse-remission process (121). MS not only leads to physical disability, but also cognitive impairment and a decline in quality of life (122), and an in-depth understanding of the pathogenesis of MS can provide new directions and ideas for its treatment. Several genetic variants, including single-nucleotide polymorphisms and mutations have been identified in complement genes (123). Previous studies have found that genetic abnormalities in the complement pathway are more likely to cause retinal neurodegeneration or increased susceptibility to visual loss in MS patients (124). A study by Vilariño-Güell analyzed the DNA of 132 MS patients and identified 12 genetic variants in genes associated with the immune pathway, providing further evidence that immunity drives MS development (125). Ingram shows that inflammation progression in the MS CNS is not dependent on infiltrating cells; inflammation can be driven by innate immune mechanisms such as complement (126). Complement activation occurs universally in MS, where complement proteins (C1q, C3) persist in MS plaques, and C1q is present in all MS plaques and plays a dominant role in the classical cascade response (126). Watkins analyzed complement expression and activation in the MS deceased organization and found C1qA expression in neurons and glial cells in the MS cortex and deep gray matter, and an increase in the upregulation and number of microglia allergenic toxin receptors in the damaged cortical gray matter area (71). C1q and C3 are deposited at synapses in the MS brain, and microglia phagocytosis of labeled synapses leads to a significant decrease in synaptic density (72). Human cadaveric samples have shown the accumulation of early complement components, including C1q, C4d, Bb, C3b-iC3b, C3d, and MAC in the cortical, hippocampal, and thalamic gray matter of MS patients (72, 127). Ramaglia et al. found a significant increase in C1q expression in the CA2 region of the hippocampus, suggesting a link between cognitive dysfunction and C1q deposition in hippocampal CA2 in MS patients (73). Interestingly, in a study by Hammond et al. using MOG _35-55_ induced experimental autoimmune encephalomyelitis (EAE), they found that C1q was not significant in causing hippocampal synapse loss and microglia activation (128). Inflammatory microglia (MIMS) and reactive astrocytes were also found in the MS nervous system. Extracellular vesicles (EVs) of astrocytes containing high levels of C1q, C3, and other complement proteins have been detected in the plasma of MS patients, which are involved in the synaptic loss process in MS (74). A positive correlation has been observed between the number density of C1q+ cells and tissue damage (129). In an experimental analysis of the spinal cord of a mouse model of chronic recurrent experimental autoimmune encephalomyelitis (crEAE), C1q expression increased throughout the disease progression phase, but complement expression decreased during the early and remission phases (75). By knocking out C1q receptors on microglia in a mouse model of MS, Absinta’s team showed that microglia proliferation indicators were largely attenuated, suggesting that C1q inhibition may be a potential way to treat MS (76). However, the Vanguri study recognized that damage to myelin phospholipids can also occur in the absence of antibodies (130). Gao demonstrated in a demyelinating mouse model that C1q may be involved in demyelination by preventing the differentiation of oligodendrocyte progenitors into mature oligodendrocytes through Wnt/β-catenin signaling activation (47).

### 4.5 Huntington’s disease (HD)

Huntington’s chorea (HD) is an autosomal dominant inherited neurodegenerative disorder (131). It is caused by an abnormally expanded CAG repeat near the N terminus of the Huntington protein gene (HTT), which produces mutant Huntington proteins upon translation(mHTT) (132). HD patients are indistinguishable from normal individuals before the onset of clinical symptoms (133). which usually start with mental changes such as personality, cognition, irritability, forgetfulness, and anxiety. As the disease progresses, motor limb incoordination appears (131, 134). HD patients are often also suicidal (135), and there is a pressing need for more tools and methods to treat this disease. Early recognition of potential suicidal ideation and symptom improvement are imperative. Complement C expression was higher in the brain of HD patients with early disease compared to controls (77), especially in the primary lesion area (136). Francis et al. suggested that C1q may co-mediate with Huntington’s protein to cause HD by inducing apoptosis in the caudate nucleus (136). Lopez-Sanchez et al. injected 3-Nitropropionic acid (NPA) into adult Wistar rats to induce an animal model of HD (137). Protein blotting and immunohistochemical analysis of brain sections from these rats showed an increase in the C3α subunit, a marker of neurotoxic A1 astrocytes, and an upregulation of cytokine IL-1α, TNFα and C1q expression in the striatum, hippocampus and cerebellum, leading to neuronal damage (78). Singhrao analyzed the complement profile of striatum in HD patients versus normal subjects and observed significant astrocyte and microglia proliferation in the caudate nucleus and internal capsule of HD, with increased complement expression, further confirmed by the increased inflammatory response in HD (79). ANX005 is a monoclonal antibody that inhibits C1q, and the safety and tolerability of ANX005 are currently being risk assessed in subjects with significant HD (NCT04514367) (134) (**Table 2**).

## 5 Relationship to inflammatory diseases in the nervous system

C1q acts as an ancient substance in the innate immune response and is naturally indispensable for its value in inflammatory diseases of the nervous system. In many diseases of the nervous system, the inflammatory response plays an important role in the pathology of neurological lesions.

### 5.1 Nerve injury

Injuries to the central nervous system, such as brain and spinal cord, trigger a robust inflammatory response, with non-adaptive immunity being the first line of defense. Numerous studies have emerged with the goal of understanding the mechanisms of nerve injury, reducing injury, improving patient outcomes, and enhancing their quality of life. Research on spinal cord injury (SCI) patients have identified polymorphonuclear leukocytes (PMNs) as the first immune cells to infiltrate the CNS following SCI, and these cells are believed to contribute to subsequent damage after CNS trauma (138). Studies have detected the expression of mRNAs encoding C1q, C3, and C4, as well as the complement proteins C1q and C3, in PMN cells in a rat model of SCI (139). Deposition of C1q was also found at the site of axon pathology and demyelination in SCI model mice (80). Moreover, C1q has been shown to mediate oligodendrocyte death, leading to demyelination and axonal loss (81). Peterson et al. found in myelin isolated from Sprague Dawley rat brain that C1q interacts with myelin-associated glycoprotein (MAG) to reduce MAG’s inhibitory effect on neurons and promote axonal growth (48). MAG is a protein associated with myelin that is produced after nerve injury and can inhibit nerve growth by binding to a common receptor complex composed of NgR, LINGO-1, and p75 ntr (140).

Traumatic brain injury (TBI) is a mechanical injury that results in rupture of brain parenchyma and blood vessels, which can be divided into primary and secondary injuries (141). The complement system promotes secondary injury in TBI, and circulating complement components can enter the brain through the injured blood-brain barrier (BBB), locally reactive microglia can activate complement-producing neurons in response to injury (142). At the site of brain injury, C1q accumulates in microglia/macrophages and neurons, and the brain can also produce a strong subsequent response to TBI by activating the local synthesis of classical and lectin complement pathway activators (82). In the TBI mouse model, it was found that the extensive connection between the thalamus and the cerebral cortex can cause secondary brain damage to the thalamus, leading to inflammation, chronic neurodegeneration, disruption of sleep spindle waves, and the occurrence of epileptic brain waves. C1q was found to accumulate around the thalamus, causing further damage through cascade reactions (83), instead, anti-C1q antibodies were used to treat TBI mice and improve their prognosis. TBI not only presents with physical motor and sensory impairment, but also has a strong correlation with the risk of dementia, especially as age increases and the degree of brain damage becomes more severe (143). Study of long-term memory deficits in aged mice with TBI may depend on the accumulation of early complement cascade components (C1q, C3, and CR3) in the brain, and inhibition of complement responses reduces cognitive impairment, and the presence of these complements may be a potential modifier of cognitive decline in the aged damaged brain (84). Manek et al. found an increased number of released microvesicles and exosome (MV/E) from human TBI cerebrospinal fluid, which is rich in cytoskeletal proteins, synaptophysin, C1q subunit B, etc. This specific MV/E causes complement activation, axonal damage, cell death and other processes (85). The level of serum C1q correlates significantly with the severity of trauma as indicated by the GCS score and the Rotterdam CT classification, and serum C1q may be a biomarker for predicting the prognosis of TBI (86). Clinical trials for complement inhibitors in TBI treatment are ongoing (NCT04489160), with promising potential for future therapies in this area (**Table 2**).

In cases of neurological injury, the complement system activation triggers an inflammatory response that worsens the injury, but complement can also attract phagocytes to promote repair and regeneration effects after CNS injury (144, 145). In a mouse model of optic nerve injury, C1q was found to play a role in nerve injury repair by interacting with microglia to phagocytose the myelin sheath of the injured optic nerve, thereby facilitating repair (87). Clinical trials examining the use of C1 inhibitors for traumatic brain injury are currently underway (NCT04489160). There are conflicting opinions on the role of C1q in nerve injury, and more research is necessary to determine how to regulate C1q in a way that benefits us.

### 5.2 Guillain-Barré syndrome

Guillain-Barré syndrome (GBS) is an acute autoimmune disease involving peripheral nerves, characterized by symmetrical progressive flaccid paralysis of the extremities, diminished neurological reflexes, and sensory abnormalities (146). GBS is mainly divided into acute inflammatory demyelinating polyneuropathy (AIDP) (which is the most common type of Grinbarism), acute motor axonal neuropathy (AMAN) and Miller-Fischer syndrome (MFS), depending on the site of onset. The etiology of the disease is still unclear, and the more authoritative factors are molecular mimicry and Campylobacter jejuni infection-related (147). Infection produces cross-reactive antibodies to human peripheral gangliosides, and complement plays important role in its pathogenesis (148). Human ganglioside GM1 antibodies accumulate mainly at the node of ranvier nerve fibers and cause their destruction. Studies on the mechanism of GM1 antibodies in model membranes have revealed that GM1 antibodies form a hexameric ring in the membrane, which can be inhibited by staphylococcal protein A binding between CH2 and CH3, and that binding of the GM1 antibody hexameric CH2 structural domain to each spherical head of C1q causes complement activation, indicating that complement is involved in an important step in the development of Grimballi syndrome disease (149). Experiments using anti-C1q antibodies in a mouse model have shown a reduction in demyelination (88). Treatment of AMAN model mice with C1q antibody can reduce the level of serum C1q and also prevent progressive development of respiratory function and neurological damage in MFS mouse model and prevent further deterioration of the disease (150). The use of anti-C1q antibodies in the treatment of GBS patients is currently being studied in clinical trials (NCT04035135)(NCT04701164) (151) (**Table 2**).

## 6 Relationship to glioma

Glioma is the most common type of brain cancer (152), glioblastoma(GBM) is the most malignant form. Currently, the standard treatment for glioblastoma involves surgical resection followed by radiotherapy and temozolomide chemotherapy (153). However, due to its rapid growth rate and poor treatment outcomes, effective management of the disease remains a challenge for clinicians seeking to improve survival time and quality of life for glioma patients. With increasing understanding of the tumor microenvironment, there is growing recognition of the role played by surface-associated proteins in tumor signaling, both between tumor cells and between tumor cells and non-tumor cells. One such protein is complement C1q, which is expressed in the human tumor microenvironment and appears to play a variety of biological roles. Recent studies have identified that complement C1q appears as an immune tolerance and immunosuppressive marker in cells of macrophage populations of healthy and tumor tissues, called tumor-associated macrophages (TAM), which can suppress cellular immunity and promote tumor growth (154, 155). Deposition of C1q and C3 was observed in tumor tissues, suggesting a role for complement in the pathogenesis of GBM (90). Complement activation has been shown to promote carcinogenesis and support the basic needs of malignant cells by maintaining proliferative signaling, angiogenesis, and anti-apoptosis, while also regulating anti-tumor immunity and promoting invasion and migration (156). There is also emerging evidence of the important role played by C1q/TNF-related proteins (CTRPs) in glioblastoma. CTRPs have been identified because they all contain a C1q globular domain, also known as the C1q/TNF superfamily, and the family includes 16 members that have been shown to have a wide range of effects on metabolism, food intake, tumor metastasis, apoptosis, vascular disease, ischemic injury, inflammation (157). Binding of the leucine-rich G protein-coupled relaxin receptor RXFP1 to C1q-tumor necrosis factor-related protein 8 (CTRP8) ligand mediates increased GBM cell migration, protein kinase C pathway activation, and lysosomal protease cathepsin B production in glioblastoma progenitor cells (158–160). Additionally, RXFP1-CTRP8 promotes actin cytoskeletal remodeling and filopodia formation through STAT3 signaling, further enhancing glioblastoma migration (89). C1q/TNF-related protein 1 (CTRP1), a member of the CTRP family, is strongly correlated with glioblastoma multiforme (GBM) and can regulate CCL2 expression, promoting tumor progression (91). Moreover, the expression level of C1q is positively correlated with different grades of glioma, and its expression may serve as a prognostic indicator. Large deposits of C1q were found in the stroma around tumor vessels, indicating that C1q may promote angiogenesis and nutrient supply to tumor cells, facilitating tumor growth (92). C1q plays a role in promoting glioma disease progression, especially through its membrane-bound form, which provides a new idea that C1q can be used as a potential target for the treatment of glioma.

## 7 Summary

C1q, as the first initiator of the classical complement response, plays an important role in the immune process in the normal physiological state, and is essential for our organism to face microbial invasion, tumor cell elimination and abnormal cell apoptosis, etc. However, if the complement regulation balance is disrupted, uncontrolled activation of C1q can lead to inflammatory and progressive damage to the host organ, generating new pathological effects, and causing various diseases. C1q plays a crucial role in neurodevelopment and neurological diseases, contributing to the development of disease progression and cognitive impairment. There are already C1q antibodies used in animal testing, and there are various types of antibody designs, and continued research is needed on how to produce the best antibodies. Additionally, regulating the activation of microglia may also provide a direction as it has been mentioned that microglia and C1q work together to produce an inflammatory effect. However, a few studies have suggested that C1q plays an active role in the repair of neurological damage, so we still need to further understand the role of C1q in the development of the disease and target interventions to fully utilize the role of C1q. In-depth exploration of the relationship between C1q and the nervous system has shown that C1q has a high potential clinical value in the diagnosis of more neurological diseases, and the amount of complement markers may be a valuable evaluation indicator for the severity and activity of the disease. As research on neurological tumors progresses, the relationship between C1q and neurological tumors continues to be confirmed, providing new directions for the treatment of neurological tumors. Although most of the current studies on the mechanism of C1q and its role in the CNS are performed in animal models, they provide many clues to the understanding of the relationship between C1q and neurological diseases, laying the foundation for the physiological functions played by C1q in the CNS and providing new ideas for the direction of treatment of related diseases. However, there is still a lack of clinical studies on C1q, its potential as a therapeutic target for neurological diseases, and the safety and efficacy of C1q inhibitors in clinical trials. C1q as a new target provides new directions for doctors and pharmaceutical companies to explore new treatment modalities, but more experiments are needed to investigate how C1q can be better applied in the clinic.

## Author contributions

WZ and YC wrote this manuscript. YC and HP revised this manuscript. All authors contributed to the article and approved the submitted version.
